# The Use of Psychotropic Medication in Iranian Children with Developmental Disabilities

**DOI:** 10.3390/ijerph18084120

**Published:** 2021-04-13

**Authors:** Roy McConkey, Sayyed Ali Samadi, Ameneh Mahmoodizadeh, Laurence Taggart

**Affiliations:** 1 Institute of Nursing and Health Research, Ulster University, Newtownabbey BT37 0QB, Northern Ireland, UK; s.samadi@ulster.ac.uk (S.A.S.); l.taggart@ulster.ac.uk (L.T.); 2 Department of Testing and Evaluation, Autism Section, Iranian Special Education Organization, Tehran 1416935684, Iran; nmahmoudizadeh@yahoo.com

**Keywords:** psychotropic medications, children, ASD, intellectual disability, management, interventions, Iran, LMIC

## Abstract

The use of psychotropic medication in children is increasing worldwide. Children with developmental disabilities seem to be prescribed these medications at a higher rate compared to their non-disabled peers. Little is known about prescribing in non-Western, middle-income studies. In Iran, the file records of 1133 children, aged 2 to 17 years, assessed as having autism spectrum disorder (ASD) or an intellectual disability (ID) in Tehran City and Province from 2005 to 2019 were collated, and information from parental reports of medications was extracted. Upwards of 80% of children with ASD and 56% of those with ID were prescribed a psychotropic medication with around one quarter in each group taking two or more medications. The rates were higher among male children showing difficult-to-manage behaviors such as hyperactivity, but less so for children of fathers with higher levels of education. The lack of alternative management strategies may be a significant driver for the use of psychotropic medications in Iran and other Low and Middle Income countries, despite their known side effects, and their failure to address the developmental needs of the children. Rather, multi-disciplinary, behavioral, therapeutic, and educational interventions are required, but these are not available widely in Iran, although a start has been made.

## 1. Introduction

International concern has been expressed about the overuse of psychotropic medication in children and particularly those with conditions such as autism spectrum disorder (ASD) and intellectual disabilities (ID) [1]. A meta-analysis of 13 studies [2] of ASD youth aged 8–11 years (totaling 96,688 participants) reported that 17.5% (95% confidence interval, 13.7% to 22.1%) received antipsychotics with a suggestion that this proportion had increased in recent years. A more recent national study in the US [3] involving nearly 8000 youth with ASD individually matched to 10 peers without ASD (*n* = approximately 80,000) found that those with ASD were over 11 times more likely to be prescribed psychotropic medication. Their use was fairly rare among pre-school children with ASD (5.7%) but far higher among school-aged children (39.0%) and adolescents (59.1%).

Risperidone was the most commonly prescribed medication across most studies and is only one of two antipsychotic medications—the other is aripiprazole—approved by the US Food and Drug Administration for use in children [4]. The other commonly prescribed drugs include fluoxetine, methylphenidate (Ritalin), clonidine, and haloperidol [2,3,4,5]. Moreover, sizeable proportions of persons with ASD were prescribed two or more medications: a median of 23% in the studies in a further systematic review with children and adults [6]. Multiple usage of psychotropic drugs is more common in older youth and adults.

There is limited evidence that medications influence the core symptoms of ASD, such as social interaction and communication, and the presence of restricted behaviors, interests, and activities [7]. Rather, the medications are aimed at addressing associated behavioral symptoms, such as irritability, tantrums, aggression, self-harm, hyperactivity, and impulsivity. Recent reviews of well-conducted randomized control trials contrasting risperidone with placebos report improvements in irritability and tantrums with some reduction in aggressive episodes and self-injurious behaviors [7,8]. Likewise, methylphenidate (Ritalin) has been well studied with improvements noted in hyperactivity and impulsivity across multiple studies, but no benefits were found for other common ASD-related behaviors, including stereotypies, repetitive behaviors, and oppositional behaviors [8]. However, there have been limited trials of other anti-psychotic medications in children and adolescents.

However, the gains from using psychotropic drugs have to be measured against the significant side effects of taking such medications. Rapid weight gain, increased appetite, fatigue, and anxiety are common with risperidone treatment of ASD. Similarly, methylphenidate has also been associated with significant adverse side effects, including decreased appetite, increased irritability, and social withdrawal [8,9]. Other psychotropic medications have not been licensed for use in children, so possible deleterious effects have not been recorded. Hence, there is a need for careful monitoring of children by experienced clinicians, such as child and adult psychiatrists and pediatricians, especially when medications are used ‘off-label’ over longer time periods [5,10].

The use of psychotropic medications in children who have intellectual disabilities has received much less attention in the literature with a focus mostly on adults. However, concerns have been expressed about the overuse of these drugs in children as a means of managing their challenging behaviors as most antipsychotic drugs are only licensed for short-term use in children of up to six weeks [11]. Although the evidence base for their use is weak, a systematic review found that risperidone and aripiprazole reduced challenging behavior among children with intellectual disabilities in the short term but with significant side effects as noted previously [12]. Little is known about the possible effectiveness of these drugs in comparison with their use in children who do not have an intellectual disability or about the interactions with other medications prescribed for children with intellectual disabilities [11].

To date, much of the research into medications and children with developmental disabilities such as ASD and intellectual disabilities has been undertaken in affluent Western countries with marked differences found between medication usage in the US and European countries [13], but with few reports of studies in low- and middle-income countries. In these countries—such as Iran—services for children with ASD and with intellectual disabilities are much less developed with few trained pediatricians, child psychiatrists, clinical psychologists, and other specialists available to provide guidance and advice to parents on how to manage their child with developmental disabilities [14]. Instead, families often rely on general physicians for diagnosing their child and helping them to manage their behaviors. A small-scale study undertaken in Tehran city in 2010–2011 with 354 children with ASD aged 7 to 14 years found that 80% reported taking at least one psychotropic medication with few differences regarding age or gender [15].

The present study aimed to examine more recent data on psychotropic medications with a younger and larger cohort of Iranian children with ASD and also those with intellectual disabilities as they rarely feature in the international literature.

### 1.1. Aims

The aims of this study were as follows:

1. To document the use of psychotropic medication in Iranian children who had a diagnosis of ASD or intellectual disability.
2. To identify the predictors associated with the use of psychotropic medication.
3. To compare children receiving polypharmacy treatment against those using a single psychotropic medication.

### 1.2. Country Context

Iran has an estimated population of 85 million people, 75% of whom live in urban areas with the capital Tehran having over 7 million. Around one quarter are aged under 15 years, but this proportion is declining as the birth rate in 2020 for women was 1.7 children [16]. The World Bank classifies Iran as having an upper-middle-income economy, but in recent years unemployment and poverty levels have risen [17].

Nearly all children (97%) are enrolled in primary education. A national screening program is undertaken for all children prior to admission to the first grade of elementary schools [18]. This mandatory program started in 1993 and is held at central locations around the country. Different professionals in the field of child development, including general physicians, nurses, and other health professionals, assess the children’s physical health, height, weight, vision, and hearing as well as administering a dental check. A trained educational evaluator examines child readiness for academic learning. Additional information is obtained from parental reports, including the child’s communication skills and behaviors, and, of particular relevance to this study, details of medication prescribed for the child are collected. Parents are requested to bring written prescriptions signed by a medical professional or general physician.

The process aims to help schools to provide for the children’s health and educational needs and to identify those with special educational needs. Those who are suspected of having developmental disabilities are referred to the Iranian Special Education Organization (ISEO) for diagnostic assessment, which is undertaken by trained psychologists and educators. The diagnosis of children with ASD is based on the Autism Diagnostic Interview—Revised (ADI-R) [19], and intellectual disabilities are assessed using the Wechsler Intelligence Scale for Children—Fifth Edition Integrated (WISC-IV) [20]. Both of these assessment tools have been translated and validated for use with Iranian children.

Young children with more severe intellectual disabilities and/or ASD are referred to centers run by the Iranian Social Welfare Organization for assessment and day care as they are deemed ineligible to attend schools.

## 2. Materials and Methods

### 2.1. Participants and Procedure

For the purposes of this study, the file records of children assessed as having ASD or ID in Tehran Province and the City of Tehran from 2005 to 2019 were collated. The rationale was two-fold. Families were more likely to have access to medical and other professionals, and also the recordkeeping was judged better in this province as the ISEO professionals had more opportunity for in-service training and supervision from senior staff. Although most of the children were currently residents of this province, subjects included children from families who had moved from other provinces and some had travelled to Tehran for the assessment.

In all, 1133 children with a diagnosis of ASD or intellectual disability were identified. Children with ASD were further divided using DSM-5 guidance and based on ADI-R assessments [21] into those with ASD Level 1 (mild impairments) and ASD Levels 2 and 3 (more severe impairments). The information contained in the children’s screening and assessment files was transferred to an Excel spreadsheet and cross-checked for accuracy. There were no missing data on the variables included in the analyses.

Table 1 summarizes the characteristics of the children and families involved in the study.

### 2.2. Data Analysis

The Statistical Package for Social Science (SPSS vers. 25: IBM, UK) was used to summarize the demographic characteristics of the children and families along with various medications they were prescribed at the time of assessment. Bivariate analyses based on chi-square tests (*p* < 0.01) were used to identify significant predictors of psychotropic medications along with a test of effect size: Cramer’s V. Discriminant analyses were used to identify the predictors that best discriminated children who received any psychotropic medication from those who had no medications prescribed and also those children who received two or more psychotropic medications (polypharmacy) from those on a single psychotropic medication.

## 3. Results

### 3.1. Medication

Table 2 summarizes the medications prescribed for the children grouped by the nature of their developmental disabilities. The differences were statistically significant with a medium to large effect size (chi sq = 85.85: df 6: *p* < 0.001: Cramer’s V = 0.195). The pattern of medication usage was similar for the children at all levels of ASD. However, many more children with ID did not receive any medication and fewer received a single psychotropic medication. The percentages receiving two or more psychotropics (Poly) and anti-convulsants were similar across all groups.

The most commonly prescribed psychotropic medications were risperidone and Ritalin, but others included memantine, fluoxetine (Prozac), clonidine, haloperidol, and paliperidone. The anti-convulsant medications included lamotrigine, sodium valproate, and carbamazepine.

In all, 38 children (3.4%) were reported to experience seizures of whom 10 (26.3%) were prescribed anti-convulsants with 25 (65.8%) taking psychotropic medication and 3 (7.9%) having no medication.

### 3.2. Predictors of Psychotropic Medication

Bivariate tests were undertaken based on the use of medication and other possible predictors, such as child and family characteristics as shown in Table 1, along with parental reports of the child’s behaviors. Of the latter, the most commonly reported were wandering (*n* = 318:28.1%), hyperactivity (*n* = 295:26.0%), aimless pacing (*n* = 201:17.7%), lack of understanding of danger (*n* = 93:8.2%), sleep problems (*n* = 91:8.0%), and aggressive behavior (*n* = 49:4.3%). However, 548 (48.4%) children showed none of these problems, 221 (19.5%) showed one behavior, 181 (16.0%) two behaviors, and 183 (16.2%) three or more behaviors.

A series of discriminant analyses were undertaken to compare the children who were prescribed psychotropic medications (*n* = 781) and no medications (*n* = 266). First, the behavior variables that had a significant relationship with the use of psychotropic medication were entered into each analysis. The behaviors that best discriminated the use of psychotropic medications were hyperactivity, aimless walking, and wandering.

Likewise, the child and family characteristics associated with psychotropic medications were included in a further discriminant analysis. The best discriminating variables were the nature of the child’s disability, father’s level of education, the child’s age, and gender.

A third discriminant analysis combined the predictors noted above. The resulting model was significant and consisted of one function (canonical correlation 0.324; Wilk’s Lambda 0.895: chi sq 115.6: df 7; *p* < 0.001). Table 3 summarizes the structure matrix of the variables that contributed significantly to the discriminant function.

The values shown in the table are similar to the variable loadings in a factor analysis. The combination of all these variables contributes to the likelihood that children will have psychotropic medication prescribed.

Children with ASD at all levels were more likely than those with intellectual disabilities to receive a psychotropic medication. A father’s level of education was the next best discriminator with children of fathers with elementary- or middle-school education more likely to have psychotropic medication prescribed. Children’s behaviors were also associated with the psychotropic prescriptions, along with males and children aged six years and over.

### 3.3. Predictors of Poly Psychotropic Medication

Comparisons could also be drawn between children who were reported to have been prescribed two or more psychotropic medications (*n* = 295) with those prescribed a single medication (*n* = 486). A similar process was followed to identify the predictors among the child’s behaviors and the child and family characteristics. The resulting model was significant and consisted of one function (canonical correlation 0.256; Wilk’s Lambda 0.934: chi sq 52.8: df 7; *p* < 0.001). Table 4 summarizes the structure matrix of the variables that contributed significantly to the discriminant function. These showed a different pattern to the previous analysis.

The use of polypharmacy treatment is most associated with children aged eight and over and with those whose disability was evident either in the first 12 months (such as those with an intellectual disability) or at three to four years of age (when ASDs become more apparent). In addition, males rather than females and those children who did not pace aimlessly were more likely to receive two or more medications as were persons with intellectual disabilities rather than those with ASD.

## 4. Discussion

Psychotropic medication is used in sizable numbers of Iranian children with intellectual disabilities and even more in those who have ASD. Although direct comparison with rates in other countries must be conducted cautiously because of sampling differences, these data suggest that they exceed those reported in the USA and Europe as noted in the Introduction and is a point to which we will return below.

The data also provide an insight into the differences between the children prescribed medications and those who were not, foremost of which was the child’s disability. This was more likely to occur in children who had a diagnosis of ASD, and this effect was present even when the behavioral characteristics were taken into account statistically. However, certain behaviors did add to the likelihood of psychotropic medications being prescribed, notably: hyperactivity, aimless pacing, and wandering, all of which can cause stress in families, especially those living in crowded apartments and houses. Yet in this sample, a father’s education—but not mother’s—added more to the discriminant function than did the behavioral indicators, in that children of fathers with higher levels of education were less likely to receive psychotropic medication. Assuming that these parents had higher incomes, factors other than the cost associated with purchasing medications need to be considered. Among the possibilities would be greater use of private therapies and pre-school education that families obtained through access to information such as books and the Internet. These advantages of more affluent families are well attested in Western countries [22].

The data also confirmed the reports in the international literature that males and older children were more likely to receive medications, although in this study, they made less of a contribution to the discriminative function than the variables noted above. However, one particular concern is the high proportion of children under six years of age receiving medication when this is discouraged internationally, not least because these drugs are not licensed for use with such young children [8].

Around one quarter of all children received two or more psychotropic medications, a figure that is similar to that reported from other studies. The discriminant analysis comparing single and multiple usage identified a different set of predictors, chief of which were the age of the child combined with the age of onset of the child’s condition. This suggests that the longer the child’s condition persists, the greater the likelihood that more than one medication will be prescribed. Other predictors were also identified, namely the increased likelihood with males and when pacing behaviors were not present, along with increased multiple use among children with intellectual disabilities. The latter finding is novel as comparative data had not featured in past research.

The number of children reported to have seizures was lower than that reported in Western studies [23,24] with just over one quarter in receipt of anti-convulsant medication. The lack of experienced diagnosticians is likely to be a major factor.

The study has a number of limitations. No information was gathered on the reasons for why the children had been prescribed the medications and the period of time the children had been taking them. Details of changes in the children’s behavior and of any side effects were not requested. It was also a cross-sectional study whereas longitudinal data on usage and changes in children would be valuable and especially necessary in Iran given the high usage of psychotropic medication revealed by these data. A wider age range might also be considered as international studies suggest higher rates of prescribing among teenagers. In addition, the present study excluded children with more severe intellectual disabilities and/or ASD who attended centers run by the Iranian Social Welfare Organization as they were deemed ineligible to attend schools. Hence, these data may underestimate the use of psychotropic medications within these populations.

The central message to emerge from this study is the likely overuse of psychotropic medication with young children in Iran compared to Western countries [2,3]. A similar pattern of overuse has been reported for the use of antibiotics in the general Iranian population as well as for other medical procedures [25]. However, a reliance on pharmacology by parents and doctors often results from inadequate or non-existent alternatives [26]. This is compounded in Iran by a lack of specialist medical expertise and a reliance on privately funded general physicians [16]. Internationally, there is widespread agreement as to the need for multi-disciplinary assessments involving psychologists, therapists, and educators as well as the use of behavioral, therapeutic, and educational interventions to nurture children’s development and support families in their care [4,11,27]. Medication may play a short-term supportive role within such interventions, but it should not be the central or sole form of intervention. A priority would be to extend the availability of these multi-disciplinary services across Iran and especially in the early years [28]. These would not only produce a better quality of life for children and families but also lead to more effective health and social care services within the country.

## 5. Conclusions

High proportions of Iranian children with ASD and ID receive psychotropic medication, including children aged five and under. This is despite the known side effects of using these medications, some of which are not licensed for use in children. The rates are higher in children who have ASD and those showing difficult-to-manage behaviors such as hyperactivity, but less so for children of fathers with higher levels of education. The lack of alternative interventions may be a significant driver for the use of psychotropic medications, but these do not address the developmental needs of the children. Rather, multi-disciplinary, behavioral, therapeutic, and educational interventions are required, but these are not available widely in Iran, although a start has been made.

## Figures and Tables

**Table 1:** The demographic characteristics of the sample (*n* = 1133).

Children		Number	%
Sex	Male	896	79.1
	Female	237	20.9
Age Groups	2–5 years	296	26.1
	6–7 years	471	41.6
	8–17 years	366	32.3
Developmental disability	ASD Level 1	702	62.0
	ASD Levels 2 and 3	90	7.9
	ID	341	30.1
Age of onset	<12 months	239	21.2
	12–23 months	175	15.5
	24–35 months	371	32.8
	36–47 months	226	20.0
	48+ months	128	11.3
Siblings	Only child	448	39.5
	One sibling	478	42.2
	Two or more	207	18.3
Family		Number	%
Mother’s age	Under 35 yrs	517	45.6
	35+ years	616	54.4
Father’s age	Under 40 yrs	546	48.2
	40+ yrs	587	51.8
Mother’s Education	Elementary	130	11.5
	Middle school	184	16.2
	High school	425	37.5
	University	394	34.8
Father’s Education	Elementary	170	15.0
	Middle school	136	12.0
	High school	372	32.8
	University	455	40.2

ASD: Autism Spectrum Disorder: ID—Intellectual Disability.

**Table 2:** The number and percentage of children receiving medication.

Medication	ASD Levels 2 and 3 (*n* = 90)	ASD Level 1 (*n* = 702)	ID (*n* = 341)
None	19 (21.1%)	109 (15.5%)	138 (40.5%)
Single psychotropic	45 (50.0%)	344 (49.0%)	99 (29.0%)
Poly psychotropics	21 (23.3%)	194 (27.6%)	80 (23.5%)
Anti-convulsant	5 (5.6%)	55 (7.8%)	24 (7.0%)

**Table 3:** The structure matrix of variables that best discriminated between children receiving psychotropic medication and the percentages within the subgroupings of the predictor variable.

Variables	Loading	% Psychotropic Medication
Type of disability	0.684	ASD Levels 2 and 3 (77.6%), ASD Level 1 (83.2%), ID (56.5%)
Father’s education	0.440	Elementary (82.8%), middle school (85.8%), high school (76.8%), university (66.9%)
Hyperactivity	0.434	Hyperactivity (85.8%), no hyperactivity (71.0%)
Aimless pacing	0.420	Paces (88.8%), no pacing (71.9%)
Age group of child	0.340	2–5 yrs (61.1%), 6–7 yrs (82.9%), 8+ yrs (75.4%)
Gender	0.334	Male (77.2%), female (65.0%)
Wandering	0.272	Wanders (81.1%), no wondering (72.1%)

**Table 4:** The structure matrix of variables that best discriminated between children receiving poly psychotropic medication and the percentages within the subgroupings of the predictor variable.

Variables	Loading	% Poly Psychotropic Medication
Age group of child	0.668	2–5 yrs (23.4%), 6–7 yrs (38.0%), 8+ yrs (46.7%)
Age of onset	0.414	Under 12 months (50%), 12–23 months (33.1%), 24–35 months (34.7%), 36–47 months (41.75), 48+ months (25.0%)
Gender	0.344	Male (39.7%), female (28.4%)
Aimless pacing	−0.312	Paces (29.3%), no pacing (39.7%)
Type of disability	0.301	ASD Levels 2 and 3 (31.8%), ASD Level 1 (36.1%), ID (44.7%)

## Data Availability

The data reported in this study are available on reasonable request from the corresponding authors.
